# The response of dual‐species bacterial biofilm to 2% and 5% NaOCl mixed with etidronic acid: A laboratory real‐time evaluation using optical coherence tomography

**DOI:** 10.1111/iej.13754

**Published:** 2022-05-06

**Authors:** Mariana Maciel Batista Borges, René J. B. Dijkstra, Flaviana Bombarda de Andrade, Marco Antonio Hungaro Duarte, Michel Versluis, Lucas W. M. van der Sluis, Xenos Petridis

**Affiliations:** ^1^ Department of Dentistry Endodontics and Dental Materials Bauru School of Dentistry University of São Paulo Bauru Brazil; ^2^ Department of Conservative Dentistry Center for Dentistry and Oral Hygiene University Medical Center Groningen University of Groningen Groningen The Netherlands; ^3^ Physics of Fluids group Technical Medical (TechMed) Center and MESA+ Institute for Nanotechnology University of Twente Enschede The Netherlands

**Keywords:** biofilm, etidronate, HEDP, irrigants, optical coherence tomography, sodium hypochlorite

## Abstract

**Aim:**

The addition of etidronic acid (HEDP) to sodium hypochlorite (NaOCl) could increase the antibiofilm potency of the irrigant, whilst maintaining the benefits of continuous chelation. Studies conducted so far have shown that mixing HEDP with NaOCl solutions of relatively low concentration does not compromise the antibiofilm efficacy of the irrigant. However, the working lifespan of NaOCl may decrease resulting in a reduction of its antibiofilm efficacy over time (efficiency). In this regard, continuous irrigant replenishment needs to be examined. This study investigated the response of a dual‐species biofilm when challenged with 2% and 5% NaOCl mixed with HEDP for a prolonged timespan and under steady laminar flow.

**Methodology:**

Dual‐species biofilms comprised of *Streptococcus oralis* J22 and *Actinomyces naeslundii* T14V‐J1 were grown on human dentine discs in a constant depth film fermenter (CDFF) for 96 h. Biofilms were treated with 2% and 5% NaOCl, alone or mixed with HEDP. Irrigants were applied under steady laminar flow for 8 min. Biofilm response was evaluated by means of optical coherence tomography (OCT). Biofilm removal, biofilm disruption, rate of biofilm loss and disruption as well as bubble formation were assessed. One‐way anova, Wilcoxon's signed‐rank test and Kruskal–Wallis H test were performed for statistical analysis of the data. The level of significance was set at a ≤.05.

**Results:**

Increasing NaOCl concentration resulted in increased biofilm removal and disruption, higher rate of biofilm loss and disruption and increased bubble formation. Mixing HEDP with NaOCl caused a delay in the antibiofilm action of the latter, without compromising its antibiofilm efficacy.

**Conclusions:**

NaOCl concentration dictates the biofilm response irrespective of the presence of HEDP. The addition of HEDP resulted in a delay in the antibiofilm action of NaOCl. This delay affects the efficiency, but not the efficacy of the irrigant over time.

## INTRODUCTION

The role of biofilms in the development and perpetuation of apical periodontitis has been established (Ricucci & Siqueira, 2010). Recently, biofilm viscoelasticity has been acknowledged as a virulent factor; it facilitates biofilm survival by influencing its response to mechanical and chemical biofilm stresses (Peterson et al., 2015). In this respect, investigating *in vitro* the capacity of endodontic irrigants to remove biofilms with certain viscoelastic features bears clinical relevance.

Biofilm removal can be assessed *in‐vitro* by means of optical coherence tomography (OCT). OCT provides quantitative measurements of biofilm removal, whilst revealing the biofilm structure at the mesoscale level (Busanello et al., 2019; Hou et al., 2019; Wagner & Horn, 2017). Indeed, OCT biofilm visualization has led to the identification of distinct biofilm layers of different cohesive and adhesive strength that seem to form when biofilms are exposed to several biocides (Petridis et al., 2019a, 2019b). This has clear clinical implications as by accumulating data on the cohesive and adhesive failure patterns of biofilms subjected to various endodontic irrigants, more effective strategies aiming at maximum biofilm removal can be devised. However, the studies conducted so far using OCT analysis have only assessed end‐point outcomes (Busanello et al., 2019; Petridis et al., 2019a, 2019b). End‐point outcomes provide a snapshot of the biofilm status at an arbitrary time point specified by the investigators. Consequently, the dynamic response elicited by the chemical and mechanical action (flow) of the irrigants is not explored at all.

Sodium hypochlorite (NaOCl) is effective against biofilms as it both kills the biofilm microorganisms and breaks down the biofilm polymeric matrix (Chávez de Paz et al., 2010; Tawakoli et al., 2017). NaOCl concentration dictates biofilm removal, as it has been shown that a short‐term static administration of 5% NaOCl enhanced both biofilm disruption and dissolution of bacterial dense biofilms compared to 2% NaOCl (Petridis et al., 2019b). NaOCl concentration determines the release of available free hypochlorite anions (OCl^−^) from NaOCl (Moorer & Wesselink, 1982). A NaOCl solution of higher concentration provides more OCl^−^ that can diffuse into the bulk biofilm and break down the glycosidic bonds present in the polymeric biofilm matrix (Tawakoli et al., 2017). A limitation of the static NaOCl administration (Petridis et al., 2019b) is that the reactive components of NaOCl are progressively deactivated when in contact with organic substrate, such as the biofilm (Baker, 1947; Haapasalo et al. 2000; Moorer & Wesselink, 1982). Thus, the conclusions drawn are considered valid only for the short time periods indicated in the studies and the clinically relevant convective interaction of NaOCl with the biofilm over time, under continuous replenishment is disregarded (Pereira et al. 2021).

Ethylenediaminetetraacetic acid (EDTA) is invariably included in root canal irrigation regimens aiming at enhanced cleanliness and disinfection of the root canal system (Basrani & Haapasalo, 2012; Zehnder, 2006). However, the loss of the active chlorine when NaOCl and EDTA interact (Grawehr et al., 2003), raises serious concerns about the dissolving and antimicrobial efficacy of NaOCl, should the irrigants be mixed during treatment (Rossi‐Fedele et al., 2012; Zehnder et al., 2005). In practice, this limits the use of EDTA as a final flush, as the alternating use of EDTA and NaOCl results in impractical irrigation protocols The use of etidronic acid (1‐hydroxyethane 1,1‐diphosphonic acid or HEDP) seems to overcome this problem. HEDP is a weak chelator that can be mixed with NaOCl without compromising the antimicrobial/antibiofilm and tissue dissolving properties of the latter (Arias‐Moliz et al., 2014; Arias‐Moliz et al., 2015; Arias‐Moliz et al., 2016; Giardino et al., 2019; Morago et al., 2016; Morago et al., 2019; Neelakantan et al., 2015; Tartari et al., 2015; Tejada et al., 2019; Ulusoy et al., 2018). In addition, continuous chelation with mixtures of NaOCl/HEDP during instrumentation results in less debris and smear layer accumulation (Paqué et al., 2012) and causes less dentine demineralization compared to NaOCl/EDTA (Tartari et al., 2018). Importantly, a non‐inferiority clinical trial has provided preliminary evidence on the lack of any adverse impact of HEDP on the clinical efficacy of NaOCl, a finding further substantiated by the microbiological results that revealed no distinct differences in the microbiota recovered from post‐irrigation samples between NaOCl and NaOCl/HEDP (Ballal et al., 2019).

Laboratory studies investigating the antibiofilm efficacy of NaOCl/HEDP mixtures have focused on the short‐term bacterial killing effects of mixtures containing relatively low NaOCl concentrations (1% or 2.5%), that are statically applied on mono‐species biofilms. Despite their undisputed value, direct translation of their findings is difficult, as clinical parameters such as irrigant flow and replenishment ought to be taken into account. In addition, the strength of mono‐species biofilms to hydrodynamic shear stresses is expected to differ considerably from multi‐species biofilms (Paramonova et al., 2009), which may skew the data on biofilm removal. Lastly, in order to examine events occurring in the entire biofilm depth and evaluating bacterial killing on the top biofilm layer, the use of OCT is more suitable than the frequently employed analysis of images acquired with confocal laser scanning microscopy.

The aim of this study was to investigate the prolonged effect of 2% and 5% NaOCl mixed with a commercial CE‐marked HEDP product for endodontic usage, on a dual‐species biofilm comprised of clinical isolates of *Streptococcus oralis* and *Actinomyces naeslundii* bacterial species. The biofilm response was expressed in terms of quantifiable biofilm disruption and dissolution, whilst the real‐time monitoring of the response allowed for the assessment of the anti‐biofilm efficiency of the solutions tested over time. The working hypothesis was that mixtures of HEDP and increased NaOCl concentration would lead to superior and faster biofilm disruption and dissolution, based on accumulated evidence indicating that EDTA, higher NaOCl concentration and extended exposure have considerable impact on this biofilm model (Busanello et al., 2019; Petridis et al., 2019a, 2019b).

## MATERIALS AND METHODS

The manuscript of this laboratory study has been written according to Preferred Reporting Items for Laboratory studies in Endodontology (PRILE) 2021 guidelines (Nagendrababu et al., 2021). The PRILE 2021 flowchart summarizes the key steps in reporting the present laboratory study (Appendix S1).

### Bacterial strains and growth conditions

Clinical bacterial isolates *Streptococcus oralis* J22 and *Actinomyces naeslundii* T14V‐J1 were streaked on blood agar plates and incubated in an aerobic incubator, at 37°C, for 24 h, and in an anaerobic incubator, at 37°C, for 48 h, respectively. A single colony was used to inoculate separate glass tubes containing 10 ml of modified brain heart infusion broth (BHI) (37 g/L BHI, 1.0 g/L yeast extract, 0.02 g/L NaOH, 0.001 g/L Vitamin K1, 5 mg/L L‐cysteine‐HCl, pH 7.3) (BHI, Oxoid Ltd.) (pre‐cultures). *S. oralis* were cultured in an aerobic incubator, at 37°C, for 24 h and *A*. *naeslundii* in an anaerobic incubator, at 37°C, for 48 h. Following, pre‐cultures were mixed with 190 ml of fresh BHI and incubated aerobically for *S. oralis* and anaerobically for *A. naeslundii* for another 16 h (main cultures). Next, bacteria were harvested by means of centrifugation (6500 *g*), with two washing steps of the bacterial pellets with sterile adhesion buffer (0.147 g/L CaCl_2_, 0.174 g/L K_2_HPO_4_, 0.136 g/L KH_2_PO_4_, 3.728 g/L KCl, pH 6.8) in between (van der Mei et al., 2008). Bacterial pellets were finally suspended in 10 ml of sterile adhesion buffer and sonicated intermittently in iced water for three times 10 s at 30 W (Vibra cell model 375, Sonics and Materials Inc.) to break bacterial chains. Bacterial concentrations were determined by counting using a Bürker‐Türk counting chamber (Marienfeld‐Superior). Both bacterial suspensions were diluted in 200 ml of adhesion buffer resulting in a dual‐species suspension containing 6 × 10^8^ bacteria/ml for *S. oralis* and 2 × 10^8^ bacteria/ml for *A. naeslundii*.

### Biofilm growth

To ensure reproducible and standardized development of bacterial cell‐dense biofilms, a constant depth film fermenter (CDFF) was used (Busanello et al., 2019; Kinniment et al., 1996; Petridis et al., 2019a, 2019b; Rózenbaum et al., 2017). CDFF was equipped with 15 sample holders. Each holder contained 5 height‐adjustable platforms. Each platform could accommodate 1 saliva‐coated dentine disk that served as the substrate for biofilm growth. Dentine disks were prepared from the crown of freshly extracted human molars. The use of extracted teeth was approved for research purposes by the Institutional Review Board of the University Medical Center Groningen. Based on the condition that the donors did not participate in any other part of the experimental protocol, the study was judged as not falling under the scope of the Medical‐Scientific Act for research with humans. A diamond‐coated core drill (6 mm, CARAT N.V.) was used to cut out dentine cylinders of 5 mm diameter, from which dentine discs of 2‐mm thickness were obtained with the aid of a water‐cooled diamond blade (IsoMet, Diamond Wafering blades 102 × 0.3 mm, Buehler) mounted in a circular cutting machine. The dentine disks were treated with 17% EDTA (Pulpdent) for 3 min for smear layer removal in a sonication bath and subsequently were autoclaved (121°C, 20 min). For the saliva coating, freeze‐dried whole saliva was used. Briefly, human whole‐saliva from 20 healthy volunteers of both sexes was collected into ice‐chilled Erlenmeyer flasks after stimulation induced by chewing Parafilm^®^ (Pechiney, Plastic Packaging) (van der Mei et al., 2008). All volunteers gave their informed consent for saliva donation, in agreement with the rules set out by the Institutional Review Board of the University Medical Center Groningen, Groningen, The Netherlands. After the saliva was pooled and centrifuged twice (10 000 *g*, 15 min, 4°C), phenylmethylsulfonyl fluoride was added to a final concentration of 1 mM as a protease inhibitor. Afterwards, the solution was centrifuged again, dialysed (24 h, 4°C) against demineralized water and freeze‐dried for storage. The lyophilized saliva was dissolved in 30 ml of adhesion buffer (1.5 g/L), stirred for 2 h and centrifuged at 6500 *g* rpm, 10°C for 5 min. The dentine discs were exposed to the reconstituted saliva under static conditions, at 4°C, for 14 h. It has to be noted that the lyophilized saliva is not submitted to any sterilization process. Saliva lyophilization does not guarantee sterilization. Nevertheless, prior to lyophilization saliva is centrifuged twice to remove any micro‐sized debris, including bacterial cells. This decreases considerably the bacterial load. After salivary protein adsorption, the substrate is inoculated with a large number of bacterial cells, which will eventually predominate over any salivary bacterial cells present on the surface. Thus, we expect none (to minimal) interference to the formation of the dual‐species CDFF biofilms (long‐term observations from studies conducted in our laboratory have never raised any concerns related to the reconstitution of the freeze‐dried saliva with buffer and the overnight saliva conditioning of the HA discs). After placing the saliva‐coated dentine discs on each platform, the height was set at 250 μm distance between the disc and the rim of the holder, thus allowing for the development of biofilms of standardized thickness (250 μm). Two hundred millilitres of the dual‐species bacterial suspension were used to inoculate the CDFF. Inoculation was performed dropwise, at a rate of 1.67 ml/min, whilst the CDFF table was slowly rotating. After inoculation, table rotation was stopped to allow for further bacterial adhesion onto the dentine discs. Finally, rotation was resumed and the biofilms were grown under continuous supply of modified BHI at a rate of 45 ml/h, at 37°C, for 96 h. Before proceeding to biofilm treatment with the irrigants, the thickness of each sample was measured with the aid of OCT. Only samples reaching the thickness of 250 μm (pre‐set height in the CDFF holders) were used in the experiment.

### Preparation of irrigant solutions

Four irrigant solutions were used to challenge the biofilm, namely, 2% NaOCl, 2% NaOCl combined with HEDP (DualRinse^®^ HEDP Medcem), 5% NaOCl and 5% NaOCl combined with HEDP. Prior to the experiments, iodometric titration was carried out to determine the concentration of the stock NaOCl solution (Sigma‐Aldrich) and the desired NaOCl concentrations were prepared by diluting stock NaOCl with demineralized water; the NaOCl solutions used in the experiments had a pH 12. Finally, for the preparation of the NaOCl/HEDP irrigant solutions, 4.5 g of DualRinse HEDP was mixed with 50 ml of either 2% or 5% NaOCl for 2 min. After mixing, the solution was drawn back into a 50‐ml polypropylene syringe with a Luer Lock opening and used immediately.

### Biofilm treatment, evaluation and outcome measures

**FIGURE 1:**
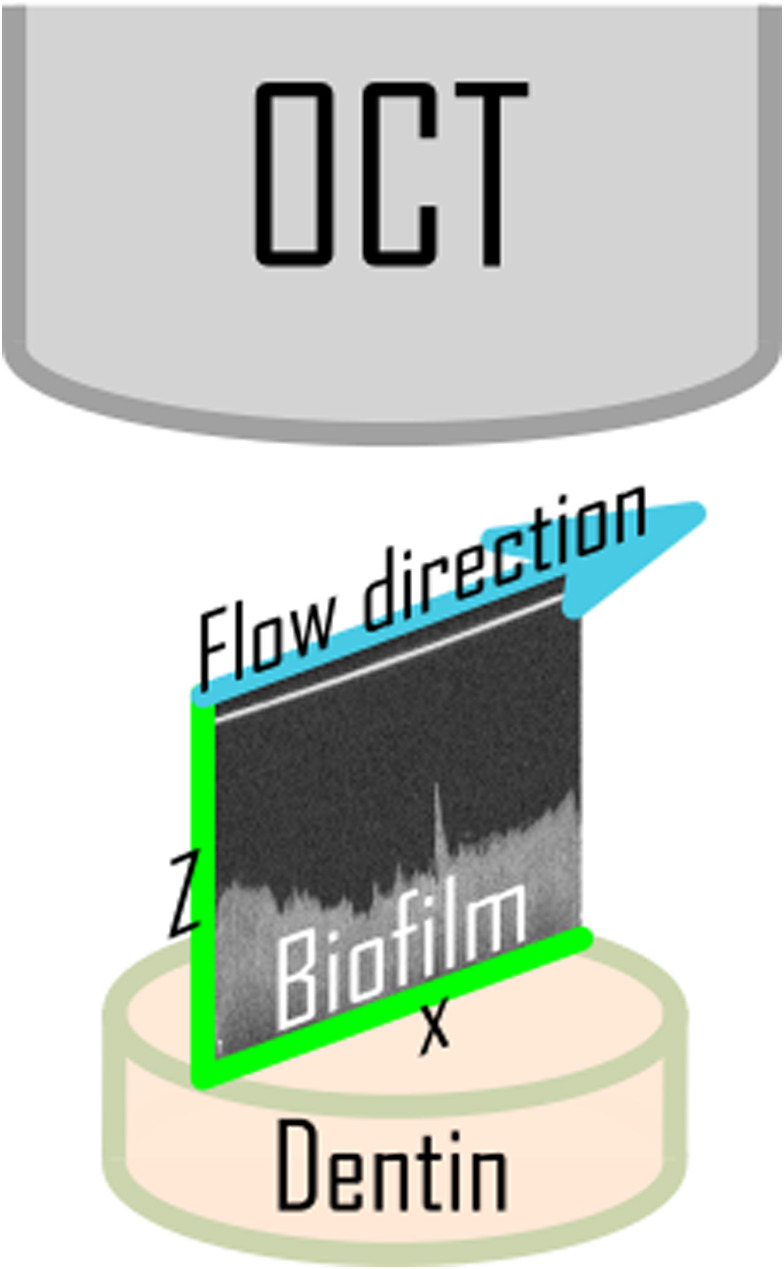
Graphical illustration of the optical coherence tomography (OCT) real‐time cross‐sectional imaging

Four experimental groups, each representing one of the irrigant solutions, were formed. Nine biofilm‐carrying dentine discs (independent samples) were submitted to treatment with each irrigant (N_per group_ = 9). Three independent experiments were carried out, during which 3 independent biofilm‐carrying dentine discs from each irrigant group were treated with the corresponding irrigant (*N*_total_ = 36). Sample size was determined based on findings from preliminary investigations and data previously published (Busanello et al., 2019; Petridis et al., 2019a, 2019b). More specifically, the following key parameters were imported in a dedicated tool used to compute statistical power analyses and required sample size (G*Power 3.1.9.7): Effect size f = 0.625 (calculated based on the minimum mean difference in the primary outcome, namely, biofilm removal set at 25% and the standard deviation SD σ within each group set at 20%), *α*‐value = .05, Power = 0.9 and Number of groups = 4. The biofilm‐carrying dentine discs were placed in a parallel plate flow chamber (PPFC), letting only the biofilm on top of the dentin disc to be exposed to the bulk of the irrigant. Irrigant was passed through the PPFC using a peristaltic pump at a flow rate of 0.05 ml/s. During irrigation, 2D real time, cross‐sectional recordings were acquired by means of optical coherence tomography (OCT, Thorlabs). Recordings were taken in 2 separate time intervals, named Phases I and II. In Phase I, the short‐term effect of the irrigant on the biofilm was recorded (0–180 s exposure). In Phase II, the long‐term effect of the irrigant on the biofilm was recorded (300–480 s exposure). The time intervals were chosen based on preliminary experiments on biofilms exposed to the plain NaOCl solutions showing a high NaOCl activity at the first 180 s that gradually decreased, until it plateaued after 480 s. Real‐time imaging was performed at 25 frames/s (1 frame taken at 0.04 s intervals), by setting the field of view at a span of 5 mm and the refraction index at 1.33. Each acquired OCT biofilm image represents a series of consecutive xz‐plane images taken along the diameter of the circular sample (the so‐called ‘OCT B‐scan’ optical cross‐section), that is a series of axial intensity profiles containing depth‐resolved structural information along the longest distance from one end of the circular biofilm‐carrying dentine disc to the other (5 mm) (Wagner & Horn, 2017) (Figure 1). Images were processed with ThorImage OCT software (Thorlabs).

The open source image processing package Fiji was used to analyse the cross‐sectional images acquired during the two phases of the OCT recordings. The image stacks, consisting of 3800 images with a resolution of 1000 × 376 pixels, were reduced to 380 measurements. This yielded a data point for every 0.4 s. A multilevel Otsu threshold was used to segment biofilm from the background (Liao et al., 2001; Otsu, 1979), as previously described (Busanello et al., 2019; Petridis et al., 2019a, 2019b). This resulted in the identification of different layers within the biofilm, namely, a layer exhibiting lower greyscale pixel intensity (that easily detaches from the bulk of the biofilm, hereafter called *disrupted layer*) and a layer exhibiting higher greyscale pixel intensity (undisturbed and firmly attached to the dentin disc, hereafter called *coherent layer*). The total number of pixels measured after background noise subtraction accounted for the total biofilm present. The number of pixels measured within the disrupted and coherent layer, which resulted after background noise subtraction and applying a multilevel thresholding, accounted for the disrupted and coherent biofilm present, respectively. The outcome measures used to assess the biofilm response to the different irrigant solutions were the following:

- Percentage total biofilm at each measurement point *t_x_*:
$$\frac{\text{Total}\;\text{biofilm}\;\text{present}\;(t_{x})}{\text{Total}\;\text{biofilm}\;\text{present}\;(t_{0}=0s)}\times 100\%$$
  where *t_0_* = 0 s is defined as the time point at which interaction between the irrigant and the biofilm takes place, namely, immediately after the introduction of the irrigant solution in the parallel plate flow chamber.
- Percentage disrupted biofilm layer at each measurement point *t_x_*:
$$\frac{\text{Disrupted}\;\text{biofilm}\;\text{layer}\;(t_{x})}{\text{Total}\;\text{biofilm}\;\text{present}\;(t_{x})}\times 100\%$$
- Rate percentage total biofilm loss (%/s), indicated by the percentage total biofilm loss over time, normalized against the starting point t_0_ = 0 s (baseline measurement).
- Rate percentage‐disrupted biofilm forming (%/s), indicated by the percentage disrupted biofilm present over time, normalized against the starting point t_0_ = 0 s (baseline measurement).
- Amount of bubbles formed during Phase I (0–180 s) and Phase II (300–480 s) (OCT slide‐by‐slide imaging). A bubble was counted as an ‘event’ when it could be visualized from the moment of its initiation until its rupture (before the end of the observation period) or when it was visible throughout the observation period.

### Statistical analysis

Statistical analysis was performed using R statistical package (version 3.6.3). Measurement points of the OCT recording were binned into 30 s intervals. Data normality were assessed with the Shapiro–Wilk test. One‐way repeated measures analysis of variance (ranova) or anova for normally distributed data and Wilcoxon signed‐rank tests or Kruskal–Wallis H for non‐normally distributed data was performed. The level of statistical significance was set at a ≤.05. Rejection of a null hypothesis, namely when *p* ≤ .05, was followed by pairwise comparisons (Bonferroni *post hoc* test for ranova, Tukey's HSD *post hoc* test for anova and Dunn's *post hoc* test for Kruskal–Wallis H).

## RESULTS

### Irrigant effect over time on biofilm removal

**FIGURE 2:**
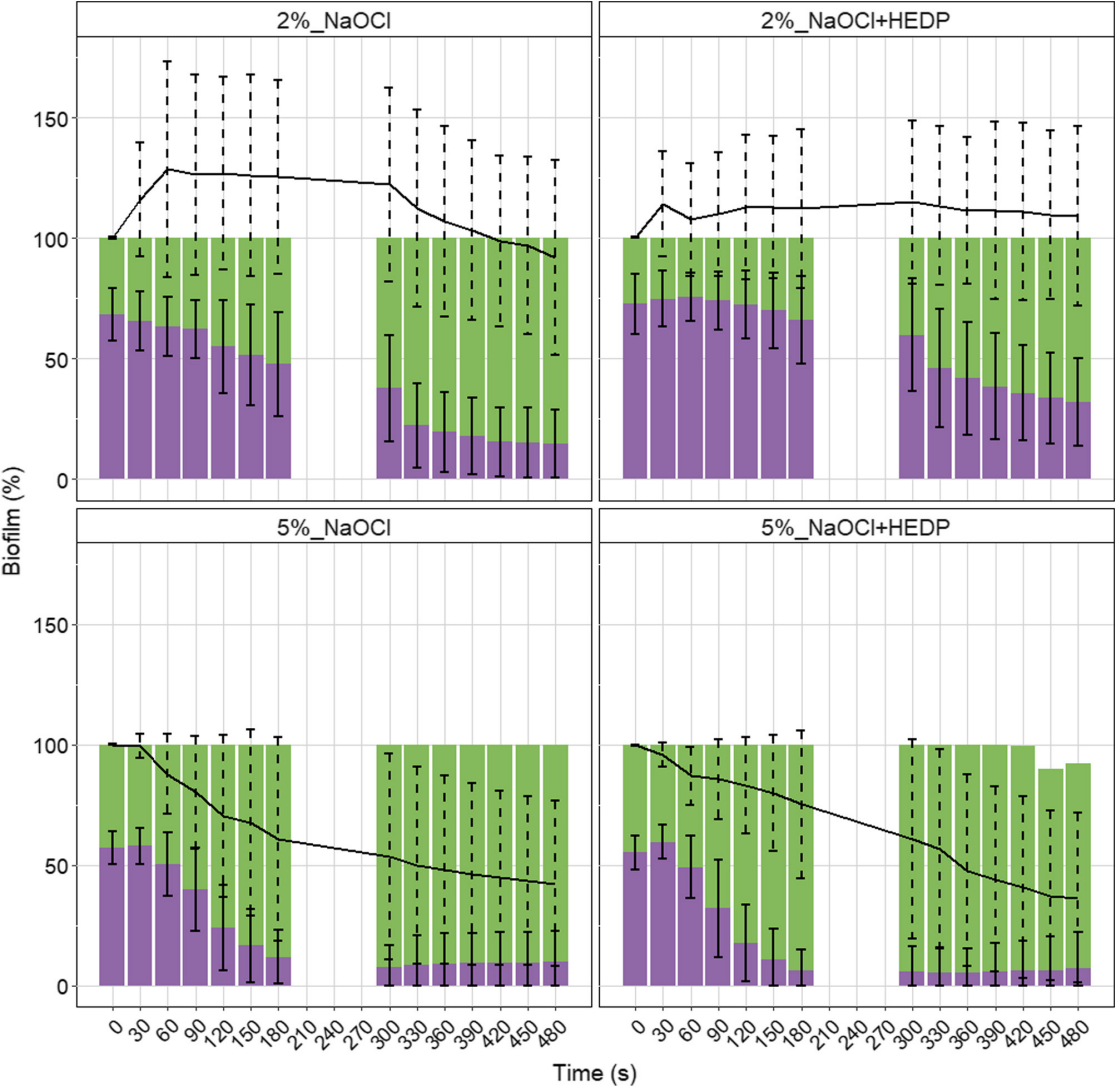
Overview of the response of the biofilm to the irrigant solutions over time. Real‐time measurement data points were binned to 30 s intervals. The black line represents the percentage total biofilm, green bars the percentage disrupted biofilm and purple bars the percentage coherent biofilm at each time interval

Biofilms exposed to 2% NaOCl demonstrated an increase in the percentage total biofilm within the first 60 s. This increase remained stable throughout Phase I, whilst a decline became evident during Phase II. The percentage total biofilm declined below the starting point of total biofilm only in the last 60 s of Phase II (Figure 2). The percentage total biofilm after exposure to 2% NaOCl/HEDP never declined below the starting point of total biofilm. On the contrary, an increase in percentage total biofilm occurred within the first 30 s of exposure, which remained stable over time (Figure 2). The percentage total biofilm after exposure to both 5% NaOCl and 5% NaOCl/HEDP showed a decline below the starting point of total biofilm at each time point measured (Figure 2). For both irrigants, a significant decline in percentage total biofilm was noted only at the end of Phase II (480 s), as compared to the starting point (Table 1). Comparing the percentage total biofilm present after the irrigation procedure, 5% NaOCl solutions, with or without HEDP, significantly decreased the percentage total biofilm in comparison to 2% NaOCl solutions, with or without HEDP. Between 2% NaOCl and 2% NaOCl/HEDP as well as between 5% NaOCl and 5% NaOCl/HEDP, no significant differences in percentage total biofilm were noted at any time point measured (Table 1).

**TABLE 1:** Percentage total biofilm present over time and comparisons within (horizontal) and between (vertical) irrigant groups

Irrigants	Exposure time (s)				
	*p*‐Values anova	0 (NS)	180 (<.01)	300 (<.001)	480 (<.001)
	*p*‐Values ranova	Percentage total biofilm (mean ± SD)			
2% NaOCl	<.01	100 ± 0	125.3 ± 40.3^A,B^	122.2 ± 40.2^a,E,F^	92.0 ± 40.5^a,I,J^
2% NaOCl/HEDP	NS	100 ± 0	112.3 ± 32.9^C,D^	114.9 ± 33.8^G,H^	109.3 ± 37.2^K,L^
5% NaOCl	<.001	100 ± 0^b^	60.7 ± 42.2^A,C^	53.6 ± 42.6^E,G^	42.4 ± 34.1^b,I,K^
5% NaOCl/HEDP	<.001	100 ± 0^c^	75.2 ± 30.5^B,D^	60.8 ± 41.3^F,H^	36.5 ± 35.1^c,J,L^

Note

One‐way repeated measures analysis of variance (ranova) with Bonferroni post‐hoc pairwise analysis (horizontal comparisons) was performed. One‐way analysis of variance (anova) with Tukey's post‐hoc pairwise analysis (vertical comparisons) was performed. Same small letters (horizontal direction) and same capital letters (vertical direction) indicate significant differences between respective groups (*p* ≤ .05).

Abbreviation: NS, non‐significant differences.

### Irrigant effect over time on biofilm disruption

For all irrigants applied, biofilm disruption was evidenced already from starting point t_0_ = 0 s (first point of interaction between irrigant and biofilm after introduction of the irrigant into the parallel plate flow chamber). Within the first 180 s, both 5% NaOCl and 5% NaOCl/HEDP had disrupted a significant amount of biofilm, which was not the case for the 2% NaOCl and 2% NaOCl/HEDP. For the rest of the observation period, no further disruption was caused by the 5% NaOCl and 5% NaOCl/HEDP. Two percent NaOCl started causing significant biofilm disruption after 300 s, whilst 2% NaOCl/HEDP elicited a significant biofilm disruption only towards the end of the observation period (480 s) (Table 2).

**TABLE 2:** Percentage disrupted biofilm layer forming over time and comparisons within (horizontal) and between (vertical) irrigant groups

Irrigants	Exposure time (s)				
	*p*‐Values anova	0 (<.01)	180 (<.001)	300 (<.0001)	480 (NS)
	*p*‐Values ranova	Percentage disrupted biofilm layer forming (mean ± SD)			
2% NaOCl	<.001	31.8 ± 11.0^a,b,A,B^	52.4 ± 21.5^c,E,F^	62.4 ± 22.0^a,I,J^	85.3 ± 14.0^b,c^
2% NaOCl/HEDP	<.001	27.3 ± 12.6^f,C,D^	34.0 ± 18.4^g,G,H^	40.2 ± 23.4^h,K,L^	68.0 ± 18.2^f,g,h^
5% NaOCl	<.001	42.8 ± 6.8^i,j,k,A,C^	88.1 ± 11.0^i,E,G^	92.1 ± 8.8^j,I,K^	90.0 ± 12.6^k^
5% NaOCl/HEDP	<.001	44.7 ± 7.1^l,m,n,B,D^	93.7 ± 8.7^l,F,H^	94.1 ± 10.5^m,J,L^	84.8 ± 25.4^n^

Note

One‐way repeated measures analysis of variance (ranova) with Bonferroni post‐hoc pairwise analysis (horizontal comparisons) was performed. One‐way analysis of variance (anova) with Tukey's post‐hoc pairwise analysis (vertical comparisons) was performed. Same small letters (horizontal direction) and same capital letters (vertical direction) indicate significant differences between respective groups (*p* ≤ .05).

Abbreviation: NS, non‐significant differences.

Comparing biofilm disruption between the irrigants applied, 5% NaOCl and 5% NaOCl/HEDP started disrupting significantly more biofilm even by the time they were introduced into the parallel plate flow chamber (0 s), keeping the same significant disruptive potential for 300 s, compared to 2% NaOCl and 2% NaOCl/HEDP. At the end of the observation period (480 s), 2% NaOCl/HEDP had caused the least biofilm disruption, without any significant difference compared to other irrigants (Table 2).

### Rate of biofilm loss

**FIGURE 3:**
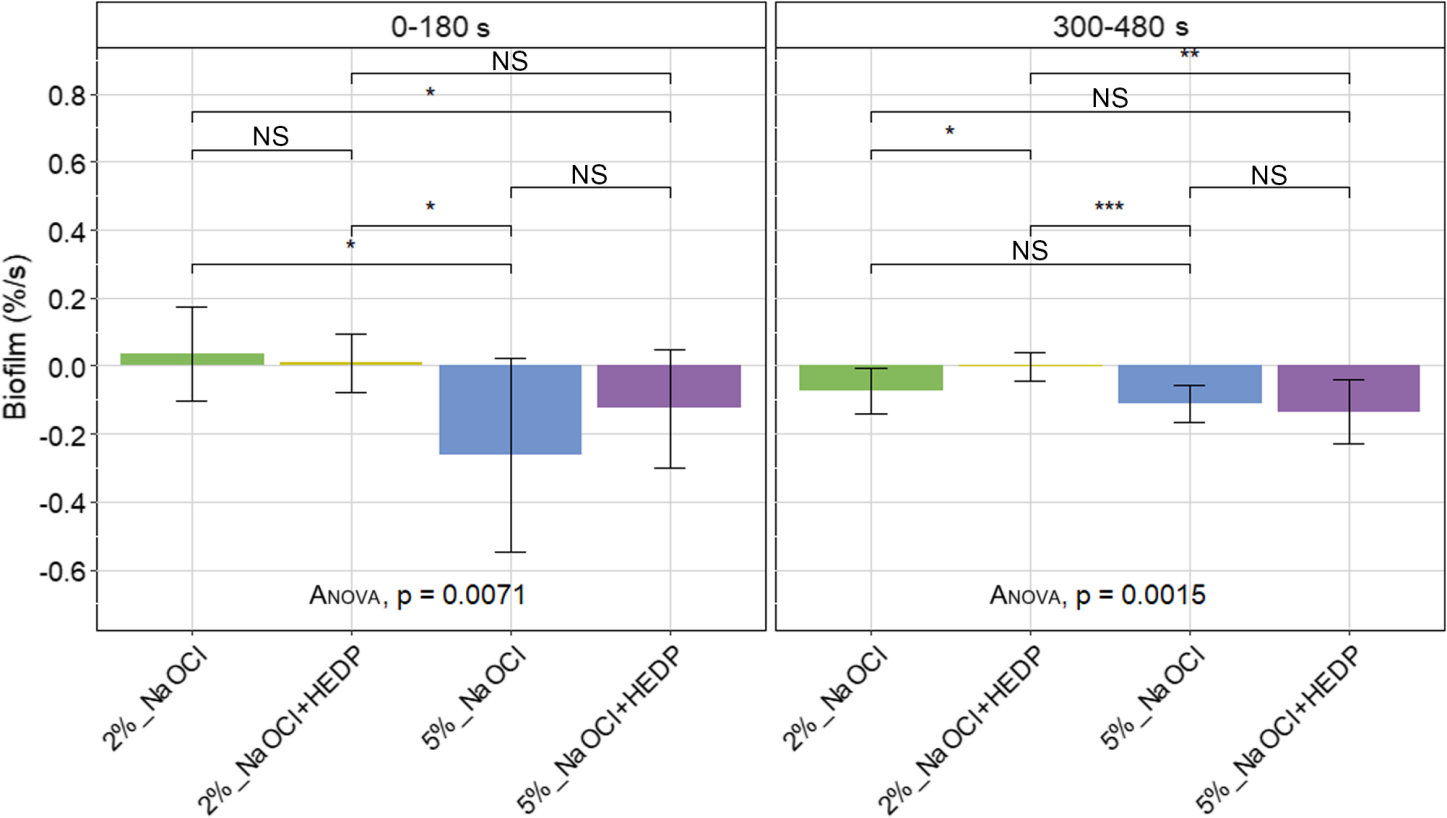
Rate percentage total biofilm loss (%/s) during Phase I (0–180 s) and Phase II (300–480 s) of exposure to the irrigant solutions. Significant differences between the irrigant solutions applied were determined using one‐way analysis of variance (anova) and indicated by * for *p* ≤ .05, ** for *p* ≤ .01 and *** for *p* ≤ .001. Non‐significant differences are indicated by NS

During the first 180 s (Phase I), 5% NaOCl decreased the percentage total biofilm the fastest, at a rate significantly higher than for 2% NaOCl and 2% NaOCl/HEDP and considerably higher than 5% NaOCl/HEDP. Five percent NaOCl/HEDP decreased the percentage total biofilm faster only when compared to 2% NaOCl during Phase I. During the last 180 s (Phase II), all irrigant solutions decreased the percentage total biofilm faster compared to 2% NaOCl/HEDP (Figure 3).

### Rate of biofilm disruption

**FIGURE 4:**
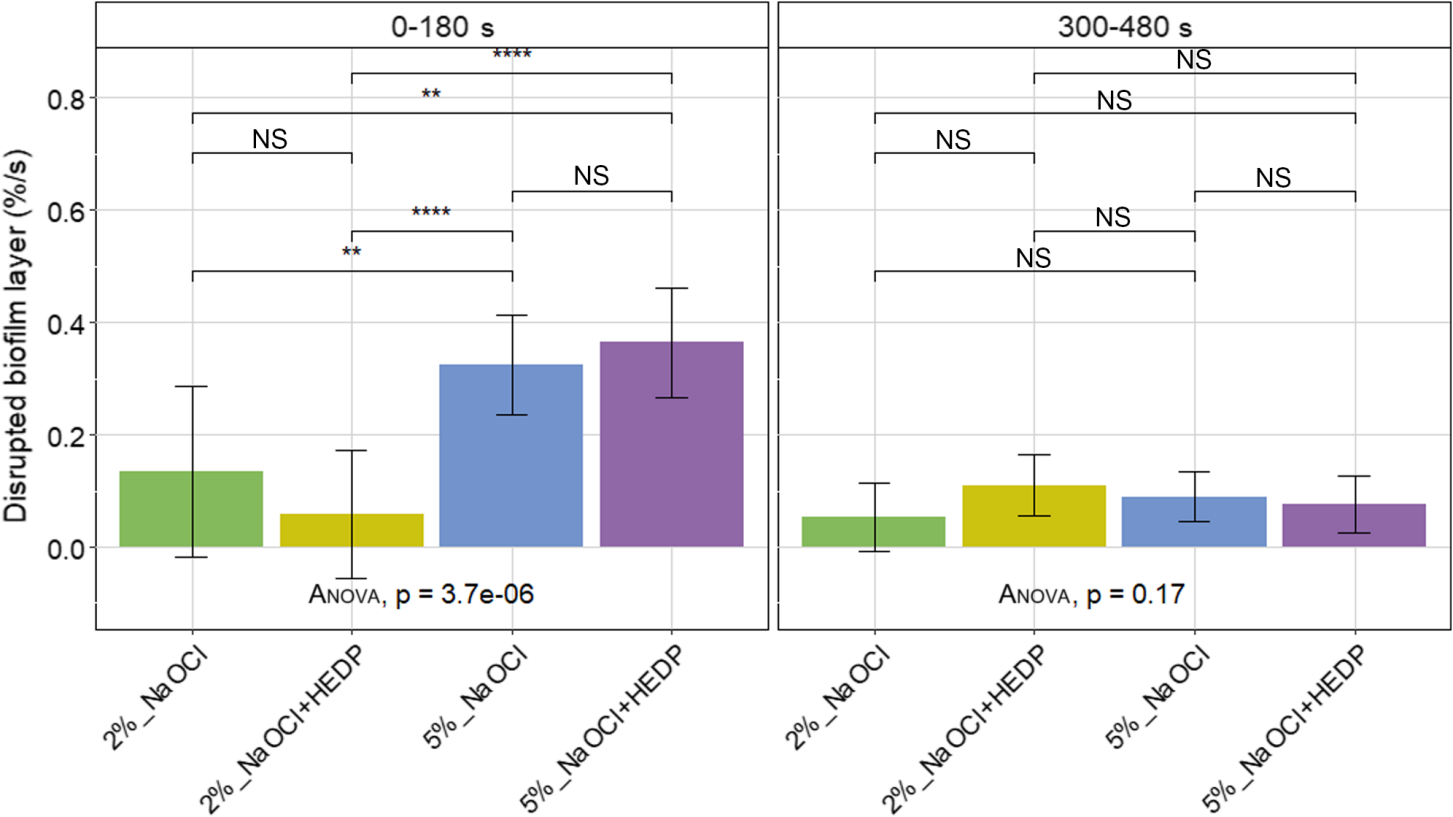
Rate percentage disrupted biofilm layer forming (%/s) during Phase I (0–180 s) and Phase II (300–480 s) of exposure to the irrigant solutions. Significant differences between the irrigant solutions applied were determined using one‐way analysis of variance (anova) and indicated by * for *p* < .05, ** for *p* ≤ .01, *** for *p* ≤ .001 and **** for *p* ≤ .0001. Non‐significant differences are indicated by NS

During the first 180 s (Phase I), 5% NaOCl and 5% NaOCl/HEDP induced biofilm disruption in a significantly higher rate compared to 2% NaOCl and 2% NaOCl/HEDP (Figure 4). During the last 180 s (Phase II), an overall decrease in the rate at which biofilm disruption occurred was noted, with all irrigant solutions disrupting biofilm at a similar, lower rate (no significant differences detected) (Figure 4).

### Bubble formation

During the first 180 s (Phase I), exposure of biofilms to 5% NaOCl and 5% NaOCl/HEDP led to a significantly higher bubble count compared to 2% NaOCl and 2% NaOCl/HEDP (Table 3). A considerable reduction in bubble count in the 5% NaOCl and 5% NaOCl/HEDP groups during the last 180 s (Phase II) was noted, with 5% NaOCl exhibiting a significant reduction compared to Phase I. Between the irrigant solutions, no significant differences were detected between the irrigant groups during Phase II. Overall, throughout the observation period, exposure of biofilms to 5% NaOCl, with and without HEDP, led to a significantly higher bubble count compared to 2% NaOCl, with and without HEDP.

**TABLE 3:** Number of bubbles formed in Phase I (0–180 s), Phase II (300–480 s) and throughout the experiment (0–480 s)

	0–180 s (Phase I)	300–480 s (Phase II)	0–480 s (Phase I & II)
2% NaOCl	0.5 (2)*^b,d^*	0 (0.75)	0 (1.25)*^f,h^*
2% NaOCl/HEDP	1 (3.5)*^c,e^*	0 (6.75)	0 (4.75)*^g,i^*
5% NaOCl	22 (19)**^a^** ^,^ *^b,c^*	1 (9)**^a^**	12.5 (22)*^f,g^*
5% NaOCl/HEDP	11 (14)*^d,e^*	6 (13.2)	8.5 (15.5)*^h,i^*

Note

To compare the number of bubbles formed between Phase I and Phase II within each group, the Wilcoxon signed‐rank test was performed. To compare the number of bubbles formed among the several irrigant groups within Phase I, Phase II and throughout the experiment, the Kruskal–Wallis H test with Dunn's post‐hoc pairwise comparison analysis was performed. Same letters in bold indicate significant differences (*p* ≤ .05) yielded by the Wilcoxon signed‐rank test, namely, pairwise comparisons between Phase I and Phase II within each irrigant (comparing in horizontal direction). Same letters in italics indicate significant differences (*p* ≤ .05) yielded by the Dunn's post‐ hoc pairwise comparison analysis, namely, pairwise comparisons among the irrigants within each Phase separately (comparing in vertical direction).

## DISCUSSION

The response of a bacterial dense dual‐species biofilm to relatively low and high NaOCl concentrations, either with or without the addition of HEDP, under laminar irrigant flow, was analysed. We showed that NaOCl concentration, irrespective of the addition of HEDP, was the driving factor of biofilm disruption and removal, enhancing efficacy and boosting the early efficiency of the irrigant. Remarkably, a slight volumetric increase (swelling) of the biofilm was observed in the first minutes following application of 2% NaOCl, with or without HEDP. On the other hand, 5% NaOCl solutions, with or without HEDP caused a significant biofilm disruption immediately after coming in contact with the biofilms. Mixing NaOCl with HEDP resulted in a delayed antibiofilm effect of the irrigant, a finding that was more prominent in the 5% NaOCl solutions. Finally, a distinct pattern of bubble formation was evident between the 2% and 5% NaOCl groups. Biofilms exposed to 5% NaOCl demonstrated a rapid growth of numerous large bubbles compared to the small‐sized and considerably less bubbles formed within the biofilms exposed to 2% NaOCl.

NaOCl was administered dynamically in an attempt to approximate the flow dynamics of root canal irrigation. Generally, *in vitro* models measure biofilm removal after ‘one‐off’ NaOCl application, thus neglecting the merit of irrigant refreshment and volume that are vital part of endodontic irrigation as this is clinically practised. In our model, a steady laminar flow was generated in a parallel plate flow chamber. The flow allowed for a more efficient transport of the NaOCl by the convective motion of the fluid (Incropera & Dewitt, 1990), ensuring refreshment, whilst retaining an adequate concentration of active OCl^−^ (Macedo et al., 2010).

In addition, the constant flow rate of 0.05 ml/s within the 8 min duration of this experiment resulted in the administration of a total volume of 26.4 ml of irrigant. This is admittedly a realistic volume of irrigant applied during a root canal treatment. Considering that monitoring and quantifying biofilm removal under constant irrigant flow rate over time, was the aim of the study, the effect of volume on biofilm removal could not be examined simultaneously and was kept constant. Given that irrigant volume plays a role in biofilm removal (Petridis et al., 2019a), we cannot exclude its confounding effect on our findings. This is an inherent limitation of the study, which could be resolved by manipulating the flow rate and measuring biofilm response at different time intervals, with the risk however of introducing additional confounding factors in the model (flow rate).

The biofilm model employed in this study fails to reproduce the geometry and configuration met in artificial or natural root canal system models. Moreover, it does not take into consideration the chemo‐mechanical process in its entirety. In that respect, the inability of a flowing irrigant solution to chemically or mechanically (shear stress) affect biofilms can be compensated to a large extent by mechanical instrumentation, irrigant agitation or the use of intracanal medicaments. Especially for mechanical debridement, its role in reducing the bacterial load is well‐established (Byström & Sundqvist, 1981; Dalton et al., 1998; Siqueira et al., 2000) and, as far as the main canal is concerned, the scraping action of the instruments will result, theoretically, in nearly complete biofilm removal (the fact that areas of the main canal will remain untouched by the instruments should not be overlooked). Instrumentation has however less impact on biofilms residing in the finest anatomical spaces and irregularities of the root canal system. Bearing in mind the limitations of this study, it should be noted that clinical extrapolation of the findings needs balanced consideration. Admittedly, testing the irrigants in root canal models bearing lateral morphological features filled with biofilms and possibly dentine debris will provide more clinically relevant findings and calls for further investigation.

Nonetheless, this biofilm model consists of clinically relevant species (Chávez de Paz et al., 2003), has a well‐defined architecture and strength (Busanello et al., 2019; Paramonova et al., 2009) and clinically relevant viscoelastic properties (He et al., 2013). These features make it suitable for studying its response under continuous, laminar flow of the irrigant solutions by means of real‐time optical coherence tomography (OCT) imaging. Taking into consideration that the viscoelastic profile of root canal biofilms is completely unknown, investigating the response of biofilms with viscoelastic features similar to oral biofilms is as clinically relevant as feasible we can get at the moment. Moreover, this model provides a reproducible *in vitro* platform for the investigation of the biofilm response to the chemical and mechanical stress resulting from the flowing NaOCl/HEDP mixture, whilst giving the opportunity to study the working mechanisms of the irrigants whilst interacting with the biofilms (Petridis et al., 2019b).

Real‐time OCT was chosen because it allows a time‐resolved assessment on the same biofilm samples, without compromising the biofilm (Busanello et al., 2019). This resolves the issue associated with biological variation that is typically encountered for individual biofilm samples. It also circumvents the limitations associated with end‐point measurements on individual biofilm samples (Petridis et al., 2019a). Perhaps more importantly, it allows us to distinguish biofilm layers, namely the coherent and disrupted layer (Busanello et al., 2019) and monitor their fate over time, under the continuous chemical and mechanical stresses exerted by a flowing irrigant.

The initial volumetric expansion of the biofilms as a response to their exposure to 2% NaOCl and 2% NaOCl/HEDP can be attributed first, to the mild anti‐biofilm action of the low NaOCl concentration that fails to remove a considerable amount of biofilm (as opposed to the 5% NaOCl concentration), and secondly to the biofilm viscoelastic properties (Busanello et al., 2019; Busscher et al., 2003; Pereira et al., 2020; Petridis et al., 2019a). Viscoelastic behaviour seems to be a trait common to biofilms that are submitted to a shear force imposed by the laminar flow. Biofilms respond dynamically to these conditions by stretching elastically without detaching (Gloag et al., 2020). This results in biofilm expansion, but not removal (Busscher et al., 2003). Interestingly, the addition of HEDP to the 2% NaOCl solution seemed to cause slightly less biofilm expansion compared to the 2% NaOCl. This was followed by biofilm retraction starting at an earlier time point (30 s instead of the 60 s noted for 2% NaOCl). Lastly, expansion remained stable, until the end point of the experiment. Based on these findings, further investigation is warranted to study the effect of HEDP on the viscoelastic properties of biofilms that are submitted to a low, but constant flow rate.

Higher NaOCl concentrations, irrespective of the presence of HEDP, removed biofilm at a higher rate compared to the less concentrated NaOCl solutions. This effect was especially prominent in the 5% NaOCl group (without HEDP) within the first 180 s, whilst slowing down thereafter. Five percent NaOCl solutions contain a higher quantity of reactive OCl^−^ compared to less concentrated solutions. As a result, an accelerated dissolution of the biofilm is expected at the first moments of their interaction (Alves et al., 2011; Cunningham & Balekjian, 1980; Gordon et al., 1981; Koskinen et al., 1980; Moorer & Wesselink, 1982; Spanó et al., 2001; Stojicic et al., 2010; Thé, 1979; Trepagnier et al., 1977). Naturally, this burst removal is followed by a decelerated organic dissolution (Moorer & Wesselink, 1982), as the reactive compound gets consumed. However, in this study, the continuous NaOCl replenishment achieved by the constant flow of new irrigant in the system compensated for NaOCl consumption. Thus, the reduced biofilm removal following the prominent biofilm removal noted in Phase 1 can be attributed to the decreasing amount of biofilm left to participate in the reaction with NaOCl.

During the last 180 s, 2% NaOCl without HEDP showed an increase in the biofilm removal rate, approaching the rate of the higher concentrated solutions. At first, this supports the idea that the assumingly compromised antibiofilm efficiency of the lower concentrated NaOCl solutions can be compensated by more frequent exchange, a larger volume and longer exposure times (Alves et al., 2011; Gazzaneo et al., 2019; Petridis et al., 2019a; Siqueira et al., 2000). However, this conclusion should be viewed with some caution as biofilm removal rate and net biofilm removal are different outcomes. Biofilm removal rate could be viewed as a measure of antibiofilm efficiency (time‐dependent effect), whilst net biofilm removal as a measure of anti‐biofilm efficacy of the irrigant (time‐independent effect). Only looking at the similar biofilm removal rates between the 2% NaOCl and the 5% NaOCl (with or without HEDP) noted towards the end of the observation period fails to recognize the fact that higher NaOCl concentrations removed considerably more biofilm and induced considerably more biofilm disruption than the lower NaOCl concentrations overall. Therefore, it may be true that continuous replenishment of lower NaOCl concentrations may compensate for their increasingly diluted antibiofilm effect as a result of the fast consumption of their reactive component whilst reacting with the biofilm, but the fact that higher NaOCl concentrations induce more pronounced biofilm disruption and removal should not be overlooked.

Previous studies have shown that combining 5% NaOCl with HEDP result in some loss of the free available OCl^−^ within the first hour after mixing, which limits the working lifespan of the solution (Biel et al., 2017; Tartari et al., 2015; Zollinger et al., 2018). As OCl^−^ is consumed when NaOCl comes in contact with biofilms (de Beer et al., 1994), the additional loss from the presence of HEDP over time could explain the extra time needed for the combined 5% NaOCl/HEDP to bring about a similar effect as the plain 5% NaOCl. In addition, the interaction between NaOCl and HEDP affects the calcium chelation ability of the latter (Biel et al., 2017). Whether and to what extent mixing HEDP with 5% NaOCl compromises the calcium complexing capacity of HEDP, thereby affecting the antibiofilm capacity of the chelator itself (EDTA, for example, shows considerable antibiofilm capacity against dense bacterial biofilms, for further information see Busanello et al., 2019) is not known and warrants further investigation. In addition, adding HEDP to the 2% NaOCl solution did not bring about any change to the rate of biofilm removal, which was remarkably low during the whole time this experiment was conducted. Again, this indicates that the chelator affected the working action of the solution, not allowing NaOCl to optimally manifest its (time‐dependent) antibiofilm efficacy.

NaOCl reacts with the proteinaceous and polysaccharidic content of the biofilm matrix (Hawkins et al., 2003; Tawakoli et al., 2017). This leads to the formation of gas bubbles filled with carbon dioxide and chloroform compounds (Mohmmed, 2017). Bubble formation is a dynamic process shown to be dictated by the reactivity between the oxidative solution and the biofilm (Petridis et al., 2019b). By taking a closer look at the biofilms within the first minutes of their interaction with the irrigants, a rapid and abundant formation of large bubbles was observed in the 5% NaOCl treatment group. The high rate of biofilm removal associated with 5% NaOCl solutions reflects the high reactivity between 5% NaOCl solutions and biofilms. In addition, the penetration capacity of 5% NaOCl is higher compared to the less concentrated NaOCl solutions (Stewart, 2003). The formation of a dense ‘bubble cloud’ increases the likelihood of coalescence of neighbouring bubbles leading to the formation of even larger bubbles. These large bubbles have increased buoyancy and, when formed deeper in the biofilm, may cause higher stresses that will eventually lead to massive biofilm structural disruption and cohesive failure. The disrupted biofilm debris is then easily removed from the bulk biofilm, for example as evidenced by the significant biofilm reduction noted after exposure to 5% NaOCl (Video S1).

When 2% NaOCl is applied, the lower reactivity between the irrigant and the biofilm does not seem to allow for large bubbles to form. Also, taking into account that these bubbles are formed in the upper biofilm layers due to the limited penetration of the 2% NaOCl compared to the 5% NaOCl, their buoyancy is initially low. Nonetheless, small bubbles also cause disruption in the biofilm structure. This disruption is associated with the structural re‐arrangement occurring naturally as a result of the volumetric expansion caused by the formed bubble. Consequently, the biofilm coherence is reduced, albeit below the critical failure level, thus similarly to what happens to a hydrogel as it swells (Macedo et al., 2014). This is evidenced by the evolution of the biofilm from a coherent visco‐elastic structure to a more viscous‐fluid like state (disrupted layer) as observed in the OCT real‐time recordings (Video S1). We hypothesize that this lies in the viscoelastic properties of the biofilms. By measuring real‐time changes occurring in biofilm mechanical properties (viscoelasticity) during the application of 2% NaOCl, validation of this hypothesis would be feasible. Towards this end, advanced OCT imaging of biofilms undergoing deformation has been shown very promising (Picioreanu et al., 2018). Ultimately, the continuous and stable administration of fresh 2% NaOCl applied in the present study, seems to have an additive effect on the reactivity of the biocide with the biofilm. This eventually leads to critical cohesive failure and biofilm removal, as evidenced between the 300 and 480 s time points.

Contrary to our working hypothesis, the continuous presence of a chemically ‘inert’ chelator did not have a synergistic effect on the antibiofilm capacity of NaOCl against CDFF biofilms. Within the limitations of this study, NaOCl concentration seems to be the driving factor that determines biofilm response (Petridis et al., 2019b). EDTA has been shown to remove biofilm more effectively compared to 2% NaOCl in a previous study employing the same biofilm model (Busanello et al., 2019). Accordingly, a superior antibiofilm effect of the combined NaOCl/HEDP irrigant solutions was anticipated. This was however not corroborated by our findings. EDTA has a higher stability constant than etidronate forming stronger bonds with metal ions (Smith & Martell, 1989; Wright et al., 2020). Nitrogen present in EDTA (CRC Press, 2017), is more likely to react with the divalent cations present in the extracellular polymeric substances (EPS) of the biofilm matrix and the receptors on the cell wall of gram‐positive bacteria, thus destabilizing biofilm matrix. HEDP is a non‐nitrogenous chelator containing phosphorous instead, which is less electronegative than nitrogen. That could explain its weaker antibiofilm action compared to EDTA. As EDTA was not used in this study, this inductive reasoning remains to be confirmed and compared to EDTA.

## CONCLUSIONS

Based on the findings and within the limitations of this study, the following conclusions can be drawn:

1. HEDP slows down the efficiency of NaOCl in terms of biofilm removal/disruption, but leads to similar results when biofilms are exposed to NaOCl/HEDP combined solutions for longer periods.
2. NaOCl concentration affects the rate of biofilm disruption and removal.
3. Bubble formation resulting from the reaction between NaOCl and the biofilm contributes to disruption of the biofilm structure and the biofilm volumetric expansion associated with the less concentrated NaOCl solutions.
4. Bubble formation parameters, such as growth rate and final size, are dependent on the concentration of the irrigant solution.
5. Optical coherence tomography is a valuable imaging tool for real‐time monitoring of interactions between reactive solutions and biofilms.

## CONFLICT OF INTEREST

The authors deny any conflicts of interest related to this study.

## ETHICS STATEMENT

All study protocols were approved by the Institutional Review Board of the University Medical Center Groningen and judged as not falling under the scope of the Medical‐Scientific Act for research with humans.

## AUTHOR CONTRIBUTIONS

Mariana Macial Batista Borges: Study conception and design, data collection, data analysis and interpretation, writing and revising the paper, final approval to the submitted version. René J.B. Dijkstra: Study conception and design, data collection, data analysis, writing and revising the paper, final approval to the submitted version. Flaviana Bombarda de Andrade: Study conception, revising the paper, final approval to the submitted version. Marco Antonio Hungaro Duarte: Revising the paper, final approval to the submitted version. Michel Versluis: Data interpretation, revising the paper, final approval to the submitted version. Lucas W.M. van der Sluis: Study concept and design, data interpretation, writing and revising the paper, final approval to the submitted version. Xenos Petridis: Study concept and design, data analysis and interpretation, writing and revising the paper, final approval to the submitted version.

## Supporting information

Appendix S1

Video S1

Supplementary Material
