# Supplementary material for: The response of dual‐species bacterial biofilm to 2% and 5% NaOCl mixed with etidronic acid: A laboratory real‐time evaluation using optical coherence tomography
Source: Int Endod J. 2022 May 6;55(7):758–71. doi: 10.1111/iej.13754 (PMC9325035; doi:10.1111/iej.13754)
Supplement: Supplementary file 3 — Supplementary Material [file IEJ-55-758-s003.docx]

**Video 1.** Real-time rendering of Phases I and II (0-3 min and 5-8 min, 6x speed) showing a 4-day mature *S. oralis* J22*/ A. naeslundii* T14V-J1 biofilm grown in a constant depth film fermenter interacting with 2% NaOCl (top left), 5% NaOCl (bottom left), 2% NaOCl/HEDP (top right) and 5% NaOCl/HEDP (bottom right), flowing at a flow rate of 0.05 mL/s (flow direction is from right to left), within a parallel plate flow chamber. The transition of the biofilm structure from a coherent to a disrupted state and the formation of bubbles within the biofilm, migrating upwards through the biofilm can be observed.

More specifically, the structure of the biofilm treated with 2% NaOCl with or without the addition of HEDP undergoes a relative slow transition from a coherent to disrupted state. During this transition, some large bubbles are formed which contribute to the reduction of biofilm volume by, occasionally, pushing chunks of biofilm into the passing irrigant. The reaction of the biofilm elicited upon the application of the 5% NaOCl solutions, with or without the addition of HEDP, is faster and more violent compared to the 2% NaOCl solutions. This results in a faster transition of the biofilm structure from a coherent to a disrupted state and the formation of more and larger bubbles, which in turn contribute to the increased biofilm removal from the surface. The increased biofilm movement observed, which can be paralleled to a viscous liquid as it moves along a surface, is an indication of the transition of the biofilm structure from a coherent to a disrupted state. The addition of HEDP to the NaOCl irrigating solutions seems to decrease the number and the size of the bubbles formed.

The white lines appearing in the videos are the result of the reflection of the top glass slide.
