# Supplementary material for: The response of dual‐species bacterial biofilm to 2% and 5% NaOCl mixed with etidronic acid: A laboratory real‐time evaluation using optical coherence tomography
Source: Int Endod J. 2022 May 6;55(7):758–71. doi: 10.1111/iej.13754 (PMC9325035; doi:10.1111/iej.13754)

PRILE 2021 Flowchart

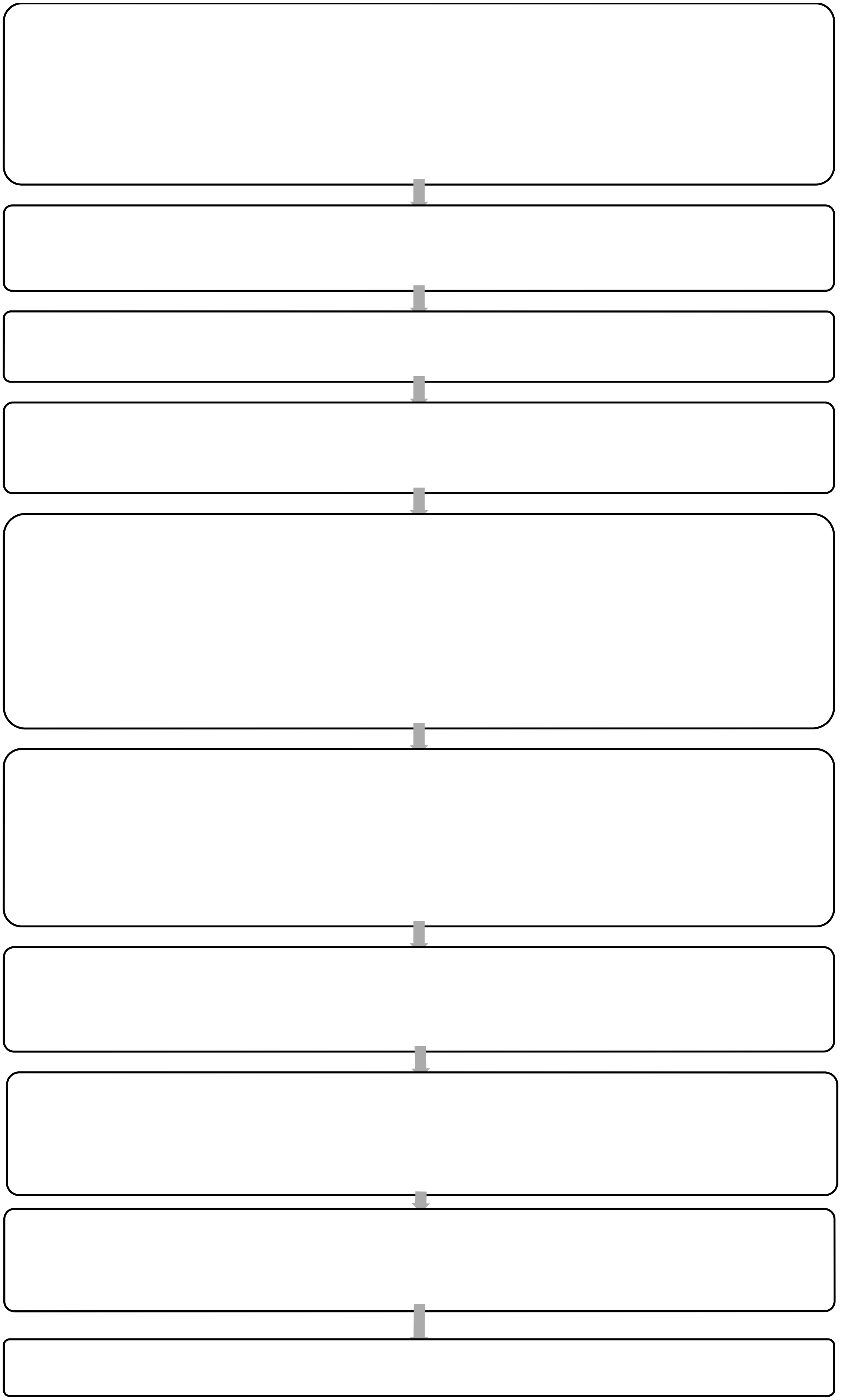

Supplement: Supplementary file 2 [file IEJ-55-758-s001.docx]
